# Polymorphisms in the α4 Integrin of Neotropical Primates: Insights for Binding of Natural Ligands and HIV-1 gp120 to the Human α4β7

**DOI:** 10.1371/journal.pone.0024461

**Published:** 2011-09-02

**Authors:** Mirela Darc, Sabrina H. Hait, Esmeralda A. Soares, Claudia Cicala, Hector N. Seuanez, Elizabeth S. Machado, James A. Arthos, Marcelo A. Soares

**Affiliations:** 1 Departamento de Genética, Universidade Federal do Rio de Janeiro, Rio de Janeiro, Brazil; 2 Programa de Genética, Instituto Nacional de Câncer, Rio de Janeiro, Brazil; 3 National Institute of Allergy and Infectious Diseases, National Institutes of Health, Bethesda, Maryland, United States of America; 4 Hospital Universitário Clementino Fraga Filho, Universidade Federal do Rio de Janeiro, Rio de Janeiro, Brazil; George Mason University, United States of America

## Abstract

The α4 integrin subunit associates with β7 and β1 and plays important roles in immune function and cell trafficking. The gut-homing receptor α4β7 has been recently described as a new receptor for HIV. Here, we describe polymorphisms of *ITGA4* gene in New World primates (NWP), and tested their impact on the binding to monoclonal antibodies, natural ligands (MAdCAM and VCAM), and several gp120 HIV-1 envelope proteins. Genomic DNA of NWP specimens comprising all genera of the group had their exons 5 and 6 (encoding the region of binding to the ligands studied) analyzed. The polymorphisms found were introduced into an *ITGA4* cDNA clone encoding the human α4 subunit. Mutant α4 proteins were co-expressed with β7 and were tested for binding of mAbs, MAdCAM, VCAM and gp120 of HIV-1, which was compared to the wild-type (human) α4. Mutant α4 proteins harboring the K201E/I/N substitution had reduced binding of all ligands tested, including HIV-1 gp120 envelopes. The mAbs found with reduced biding included one from which a clinically-approved drug for the treatment of neurological disorders has been derived. α4 polymorphisms in other primate species may influence outcomes in the development and treatment of infectious and autoimmune diseases in humans and in non-human primates.

## Introduction

Integrins are essential molecules involved in a variety of immunomodulatory functions in vertebrates, including cell adhesion, cellular trafficking and immune responses [1]. They function as heterodimeric receptors that mediate adhesion to immunoglobulin superfamily molecules and to extracellular matrices. Twenty-four different integrin heterodimers are currently recognized, formed by combination of at least 18 α-subunits and 8 β-subunits, each one encoded by a different gene [2]. Specific integrin expression is found in distinct cell types and the presence of integrins on the cell surface plays a key role in the migration of cells to different tissues. In addition to their physiological role, integrins are increasingly recognized to function as receptors for many viruses, including rotaviruses, herpesviruses and retroviruses such as HIV [3], [4], [5], [6]. Invariably, viruses bind to integrins through the same domains as their natural ligands, by mimicking immunoglobulin binding motifs.

The α4 integrin (CD49d) is encoded by the *ITGA4* gene (geneID 3676), located in chromosome 2 at 2q31.3. It comprises 28 exons, spanning over 80 kb. The α4 subunit binds to either β1 or β7 subunits to form heterodimeric integrin receptors [7]. α4 is highly expressed on T and B lymphocytes, monocytes, natural killer and dendritic cells [7], [8]. In primates, the heterodimer α4β7 acts as a gut homing receptor, targeting and binding α4β7-expressing cells to mucosal addressin cell adhesion molecule-1 (MAdCAM-1) on capillary venules. α4β1, on the other hand, induces mesenchymal cell migration and B- and T-cell development by binding preferentially to fibronectin and vascular cell adhesion molecule-1 (VCAM-1) [1], [8]. α4β7 and α4β1 adopt three conformations that exhibit different affinities for MAdCAM and VCAM: inactive, intermediate and extended/activated [9]. The conversion between these forms relies on conformational changes that the heterodimer is subject to in response to a complex set of signals that includes ligand binding.

Recently, the gut homing receptor α4β7 has been recognized as a receptor for HIV-1, a binding governed by a tripeptide in the V2 loop of the viral gp120 that mimics the structure present in the integrin natural ligands [4]. As a consequence, HIV-1 gp120 binds to the same integrin domains defined as the target motifs to MAdCAM-1 and VCAM-1 [4], [10], which correspond to epitopes encoded by *ITGA4* exons 5 and 6. It has been suggested that such binding facilitates the targeting of HIV-1-infected T-lymphocytes to the gut-associated lymphoid tissue (GALT), where a massive depletion of CD4^+^ T-cells occurs, leading to the HIV-1-induced immune dysfunction observed during virus acute infection [4]. HIV gp120 also appears to bind differently to the distinct conformational forms of α4β7 [4]. The interaction between lentiviruses and α4β7 is reiterated in another pathogenic model of lentiviral infection, that of simian immunodeficiency virus (SIV)-infected rhesus macaques [11], [12], [13]. Consistent with this model, recent evidence has been presented which indicates that blocking α4β7 during acute infection of rhesus macaques with SIV reduces plasma- and GALT-associated viral replication [14].

An exception to the Primates order, New World primates (NWP) are not reported to be infected *in natura* or in captivity by SIV. Several host genes encoding proteins that counteract lentivirus infection, collectively called restriction factors, have been studied in NWP, and diverse genus- and species-specific restriction phenotypes have been described for this primate group. These restriction factors include CCR5 and CXCR4 [15], [16], [17], TRIM5α [18], [19], [20], [21], [22], members of the APOBEC gene family [23], [24] and tetherin [25]. We hypothesized that genetic determinants in the *ITGA4* gene, translated into nonsynonymous polymorphisms in the α4 subunit of α4β7, can also contribute to the plethora of restriction phenotypes that render NWP resistant to lentiviral infections. With this objective, we analyzed genetic polymorphisms of *ITGA4* in a large, representative collection of NWP. We found several new *ITGA4* variants with synonymous and non-synonymous substitutions. Functional analyses of some of these variant α4 integrins indicate impaired affinity to natural ligands, to α4β7-directed monoclonal antibodies and to various HIV-1 gp120 molecules. Some of these changes may explain, at least in part, the restriction of some NWP species to lentivirus infection. Our study has also provided detailed information on the interaction between the α4 integrins to their natural ligands as well as to HIV gp120.

## Materials and Methods

### Animal sources and genomic DNA

Genomic DNA of 164 samples of New World primates previously available at the Brazilian Cancer Institute (INCA) comprising all three Platyrrhini families (Atelidae, Cebidae and Pitheciidae), 15 genera and 48 different species were analyzed. In addition, multiple specimens (ranging from 2 to 19) from a single species were included (Table 1). Original samples have been previously collected by venous puncture, and all procedures were carried out following the national guidelines and provisions of IBAMA (Instituto Brasileiro do Meio Ambiente e dos Recursos Naturais Renováveis, Brazil; permanent license number 11375-1).

**Table 1:** New World primate specimens analyzed in this study.

Species name	Common name	No. of specimens
**Family Atelidae**		
* Alouatta belzebul*	red-handed howler monkey	6
* Alouatta caraya*	black howler monkey	5
* Alouatta guariba*	red-and-black howler monkey	2
* Alouatta seniculus*	red howler monkey	2
* Ateles paniscus*	black spider monkey	1
**Family Cebidae**		
* Brachyteles arachnoides*	southern muriqui	3
* Aotus sp.*	owl monkey	6
* Aotus azarae*	southern owl monkey	20
* Aotus infulatus*	feline night monkey	1
* Callimico goeldii*	Goeldi's marmoset	3
* Callithrix aurita*	white-eared marmoset	2
* Callithrix geoffroyi*	Geoffroy's marmoset	3
* Callithrix jacchus*	white-tufted-ear marmoset	2
* Callithrix kuhlii*	wied's marmoset	3
* Callithrix penicillata*	black-pencilled marmoset	4
* Cebuella pygmaea*	pygmy marmoset	2
* Cebus sp.*	capuchin monkey	6
* Cebus albifrons*	white-fronted capuchin	5
* Cebus apella*	brown-capped capuchin	19
* Cebus capucinus*	white-faced sapajou	1
* Cebus cay*	hooded capuchin	1
* Cebus olivaceus nigrivittatus*	weeper capuchin	5
* Cebus xanthosternos*	yellow-breasted capuchin	5
* Leontophitecus chrysomelas*	golden-headed lion tamarin	4
* Leontophitecus chrysopygus*	gold-and-black lion tamarin	3
* Leontophitecus rosalia*	golden lion tamarin	3
* Mico argentata*	silvery marmoset	5
* Mico emiliae*	snethlage's marmoset	6
* Mico humeralifer*	tassel-eared marmoset	3
* Mico melanura*	black-tailed marmoset	2
* Saguinus bicolor*	pied bare-faced tamarin	1
* Saguinus fuscicollis*	brown-headed tamarin	2
* Saguinus imperator*	emperor tamarin	3
* Saguinus martinsi*	Martin's tamarin	1
* Saguinus midas*	golden-handed tamarin	3
* Saguinus mystax*	moustached tamarin	2
* Saguinus niger*	black-handed tamarin	1
* Saguinus oedipus*	cotton-top tamarin	1
* Saimiri sp.*	squirrel monkeys	4
**Family Pitheciidae**		
* Cacajao melanocephalus*	black-headed uakari	3
* Callicebus sp.*	titi monkeys	2
* Callicebus coimbrai*	Coimbra-filho’s titi monkey	1
* Callicebus donacophilus*	bolivian titi	1
* Callicebus hoffmansi*	Hoffmann's titi	1
* Callicebus moloch*	dusky titi monkey	3
* Callicebus nigrifrons*	black-fronted titi monkey	3
* Callicebus personatus*	masked titi	3
* Chiropotes sp.*	bearded saki	3
* Chiropotes albinasus*	red-nosed bearded saki	1
* Chiropotes israelita*	brown-backed bearded saki	1
* Chiropotes satanas*	black-bearded saki	1

### PCR amplification and sequencing of *ITGA4* gene exons 5 and 6

Genomic DNA was extracted from specimens’ peripheral blood mononuclear cells using the QIAGEN Genomic DNA extraction kit (QIAGEN, Chatsworth, CA), according to the manufacturer's specifications. A PCR reaction was conducted to amplify a *ITGA4* genomic region comprising exons 5 and 6 (with the intervening intron 5), a fragment of approximately 1,550 bp. Primers were designed according to the human *ITGA4* gene sequence publically available in the GenBank database (acc. # NW_921585) with the following sequences: ITGA4-F (sense) 5′GTTTAATATTTCATTTTA-3′ (nucleotides 21843–21863) and ITGA4-R (antisense) 5′-CAGACATGATGCAGATGTTGCAC-3′ (nts 23374–23394). PCR conditions were in the presence of 100–200 ng DNA, 5 µl 10X PCR buffer, 0.4 µl 25 mM dNTP mix, 25 pmol of each primer, 2 µl of 25 mM MgCl_2_ and 0.4 µl of 5 UI/ ml *Taq* Platinum DNA polymerase (Life Technologies, Carlsbad, CA). Reactions were carried out in a *Veriti*® Thermal Cycler (Applied Biosystems Life Technologies) with an initial denaturing step of 94°C for 2 min, followed by 35 cycles of 94°C for 30 sec, 46°C for 30 sec and 72°C for 2 min. A final cycle of extension at 72°C for 8 min followed each reaction, to complete unfinished DNA strands. A second PCR (semi-nested) was then carried out to individually amplify exons 5 and 6. For exon 5, primers ITGA4-F (sense) and ITGA4-RI (antisense; 5′-GGTACTATAAAAATTGACAAAC-3′; nts 22027-22049) were used. For exon6, ITGA4-FI (sense; 5′-CAGGATTTAATTGTGATGGG-3′; nts 23241-23260) and ITGA-R (antisense) were employed. PCR reactions were carried out with 5 µl of the initial PCR reaction described above and under the same conditions, with the exception of the extension time, set to 30 sec.

**Figure 1:**
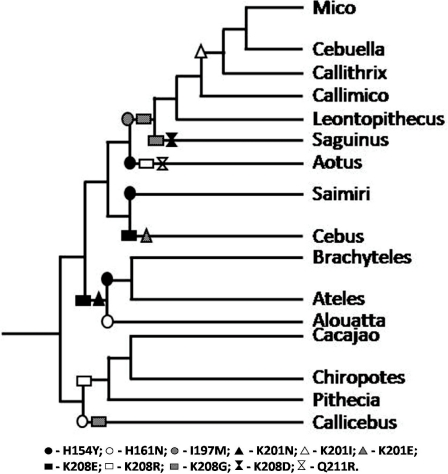
Representation of the likely emergence of α4 amino acid substitutions encoded by *ITGA4* exons 5 and 6 across neotropical primate radiation. The consensus amino acid sequence of the Platyrrhini group was used as the ancestral root of the tree, and residue replacements refer to that sequence. Each replacement had its emergence estimated and placed into the most updated Platyrrhini phylogeny according to Perelman *et al*. [29].

PCR products corresponding to exons 5 and 6 of individual specimens were purified with the Illustra™ GFX PCR DNA and Gel Band Purification kit (GE HealthCare, São Paulo, Brazil), quantified and sequenced using the Big Dye v.3.1 kit (Life Technologies, Carlsbad, USA). Sequencing primers used were the same as in the 2^nd^ round PCR reaction described above. DNA sequencing was carried out in an automated ABI 3130XL Genetic Analyzer (Life Technologies) and manually edited with the software SeqMan v7.0 (DNASTAR Inc, Madison, USA). Sequences were then aligned using BioEdit v7.0 [26], and a consensus sequence for each species was generated. For means of comparison, *ITGA4* sequences from human (*Homo sapiens*), chimpanzee (*Pan troglodytes*), rhesus macaque (*Macaca mulatta*), mouse (*Mus musculus*), horse (*Equus caballus*), cow (*Bos taurus*) and African green monkey (*Chlorocebus sp*.), retrieved from GenBank, were also included in the alignment. Deduced amino acid sequences were generated from each DNA consensus sequence and compared between species. Amino acid substitutions characteristic of each neotropical primate genus were placed in the currently accepted Platyrrhini phylogenetic tree (Figure 1) to estimate their appearance during Platyrrhini radiation.

All DNA sequences generated in this work were submitted to the GenBank nucleotide sequence database and were assigned the accession numbers JF938225 to JF938528.

### Site-directed mutagenesis and expression of mutated α4 subunits

Non-synonymous polymorphisms found in the newly characterized *ITGA4* alleles of NWP were inserted into a mammalian expression plasmid containing the cDNA encoding the reference published human *ITGA4* gene, in an individual fashion or in different double, triple or quadruple combinations, that included the five polymorphisms found in some specimens. Site-directed mutants were constructed using the QuickChange^TM^ Site-directed mutagenesis kit (Agilent Technologies, Inc., Santa Clara, CA), according to the fabricant's specifications. A complete list of the mutants generated in this study, and the representative species in which the polymorphisms were observed, is found in Table 2. Table S1 depicts all primers used in the site-directed mutagenesis experiments, together with the annealing temperatures employed. Mutant plasmids were scaled and all mutations were further confirmed by DNA sequencing to confirm the presence of the desired mutation(s) and the lack of additional changes.

**Table 2:** Single and multiple α4 mutants generated in this study.

MUTANTS		α4 AMINO ACID POSITION(S)	NWP genus or family observed
Single	*ITGA4* Exon 5	Y154H	*Alouatta*
		N161H	*Brachyteles, Aotus*
	*ITGA4* Exon 6	K201I	*Callithrix, Mico*
		K201E	*Cebus*
		K201N	*Alouatta, Ateles, Brachyteles*
		K208E	*Alouatta, Cebus*
		K208G	*Callithrix, Cebuella, Leontopithecus, Mico, Callicebus*
		Q211R	*Aotus*
Double		Y154H / N161H	Cebidae, *Chiropotes*
		Y154H / K208N	*Alouatta*
		N161H / K208I	*Callithrix, Mico*
		N161H / K208R	*Aotus, Chiropotes, Pithecia*
		K201N / K208E	Atelidae
Triple		Y154H / N161H / K208I	*Callithrix, Cebuella, Mico*
		Y154H / N161H / K208E	*Cebus*
		Y154H / K201N / K208E	*Alouatta*
		N161H / K201N / K208E	*Ateles, Brachyteles*
Quadruple		Y154H / N161H / I197M / K201I	*Callithrix, Cebuella, Mico*
Quintuple		Y154H / N161H / I197M / K201I / K208G	*Callithrix, Cebuella, Mico*

### Cell culture, transfections and α4β7 expression and detection

293T cells [27] were maintained in DMEM supplemented with 10% FBS and 2% penicillin/streptomycin (all reagents from Life Technologies) in 75 cm^2^ cell culture flasks at 37°C in a 5% CO_2_ incubator. On the day prior to transfections, 5×10^5^ cells were split into 100 mm^2^ tissue culture plates (Tecno Plastic Products AG, Trasadingen, Switzerland) with 10 ml of supplemented DMEM, and incubated overnight as above. On the following day, plates were transfected (PolyFect® Transfection Reagent (QIAGEN, Valencia, CA) in replicates with an expression plasmid harboring a cDNA copy of the wild-type β7 subunit [4], together with one of the α4 subunit mutants. Co-transfection of wild-type α4 and β7 was carried out in parallel as a control. At 48 h post-transfection, cells were harvested with versene, rinsed thoroughly and stained for flow cytometry analysis.

### α4β7 binding efficiencies of antibodies / ligands

Cells were stained with phycoerythrin-conjugated monoclonal antibodies (mAbs) using standard procedures as described in Arthos *et al*. [4] mAbs used were 7.2, 2b4 and HP2/1 (anti-α4); FIB 504 (anti-β7); ACT-1 (anti-α4β7 heterodimer); all obtained from Serotek (Oxford, UK) or Millipore (Billerica, MA), except for ACT-1, available at the NIAID AIDS Reagent Program. The 7.2 anti-α4 binds to a distal epitope relative to the natural ligand binding site on α4 [10], and was thus used a control for the levels of both mutant and wild-type α4 expression on the cell surface. Cells were also stained with recombinant MAdCAM- and VCAM-Ig fusion proteins (R&D Systems, Minneapolis, MN), as described in Arthos *et al*. [4] Finally, α4β7-transfected cells were also tested for HIV-1 gp120 binding using recombinant gp120 proteins biotinylated by amine-coupled chemistry as previously detailed [4]. The following recombinant viral gp120's were tested: Q23.335 (subtype A; GenBank acc. # DQ136335), 93MW959 (subtype C; GenBank acc. # U08453), and a derivative of AN1 (subtype B, sequence available at http://ubik.mullins.microbiol.washington.edu/HIV/Doria-Rose2005/) harboring a N201Q substitution that enhances binding to α4β7 [28]. All gp120's tested were CCR5-tropic. Binding efficiencies (BE) of antibodies and recombinant proteins to α4β7-transfected cells were represented as the ratio of the MFI of α4β7-transfected cells compared to the MFI of mock-transfected cells. Whenever multiple experiments were available, data were represented as the average BE of different experiments, and the associated standard errors are provided. For these cases, the BE to different α4 variants were compared with Student's *t* tests and *p*-values ≤0.05 were considered significant.

## Results

### Analysis of *ITGA4* genotypes in New World primate specimens

**Figure 2:**
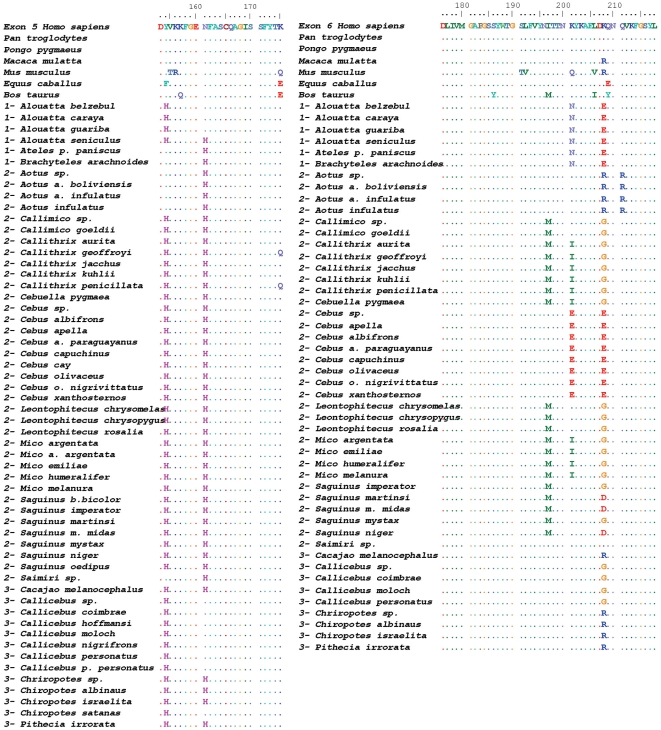
Amino acid alignment of the predicted sequences encoded by ITGA4 exons 5 (left) and 6 (right) of representative species of neotropical primates. Sequences of each species represent a consensus from 2-19 individual specimens sequenced in this study. Residue numbering corresponds to that of the mature α4 subunit protein, after cleavage of the signal peptide. Dots represent identities. Numbers before species’ names depict Platyrrhini families as follows: 1- Atelidae; 2- Cebidae; 3- Pitheciidae (as proposed by Schneider *et al*. [38]). For means of comparison, *ITGA4* sequences from human (*Homo sapiens*), chimpanzee (*Pan troglodytes*), rhesus macaque (*Macaca mulatta*), mouse (*Mus musculus*), horse (*Equus caballus*), cow (*Bos taurus*) and African green monkey (*Chlorocebus sp*.) are shown at the top of the alignment.

The analysis of *ITGA4* gene exons 5 and 6 from over a hundred specimens of neotropical primate species showed a multitude of different genotypes. Of note, in no case were the exon 5 and 6 sequences obtained from these specimens identical to that of either human *ITGA4* (GenBank acc. # NM_000885) or the Old World primate specimens we analyzed. Numerous non-synonymous changes were found in distinct specimens, but most changes occurred at 5 codons of the sequences analyzed: codons 154 and 161 in exon 5, and codons 197, 201 and 208 in exon 6 (codon numbering refers to the mature α4 protein, after processing of its signal peptide) (Figure 2 and Table 2). At these positions, the major polymorphisms found were N161H, Y154H, I197M, K201N/I/E and K208E/R/G. Most polymorphisms were found individually or in combination within specific clades of NWP (genera or families; Table 2), characterizing ancestral polymorphism arisen during Platyrrhini radiation. Based on a recently updated Platyrrhini phylogeny [29], we hypothesize that these variants emerged from a common ancestral *ITGA4* during Platyrrhini evolution (Figure 1).

The nature of the amino acid changes characteristic of *ITGA4* polymorphisms varied significantly. Whereas both substitutions in the coding sequence of exon 5 (positions 154 and 161) and the I197M substitution in exon 6 did not involve a change the electrostatic charge, K201I and K208G in exon 6 did involve the loss of a positively-charged amino acid, resulting in change in the local electrostatic environment in this region of α4.

### Binding properties of NWP α4 variants to monoclonal antibodies and natural ligands

**Figure 3:**
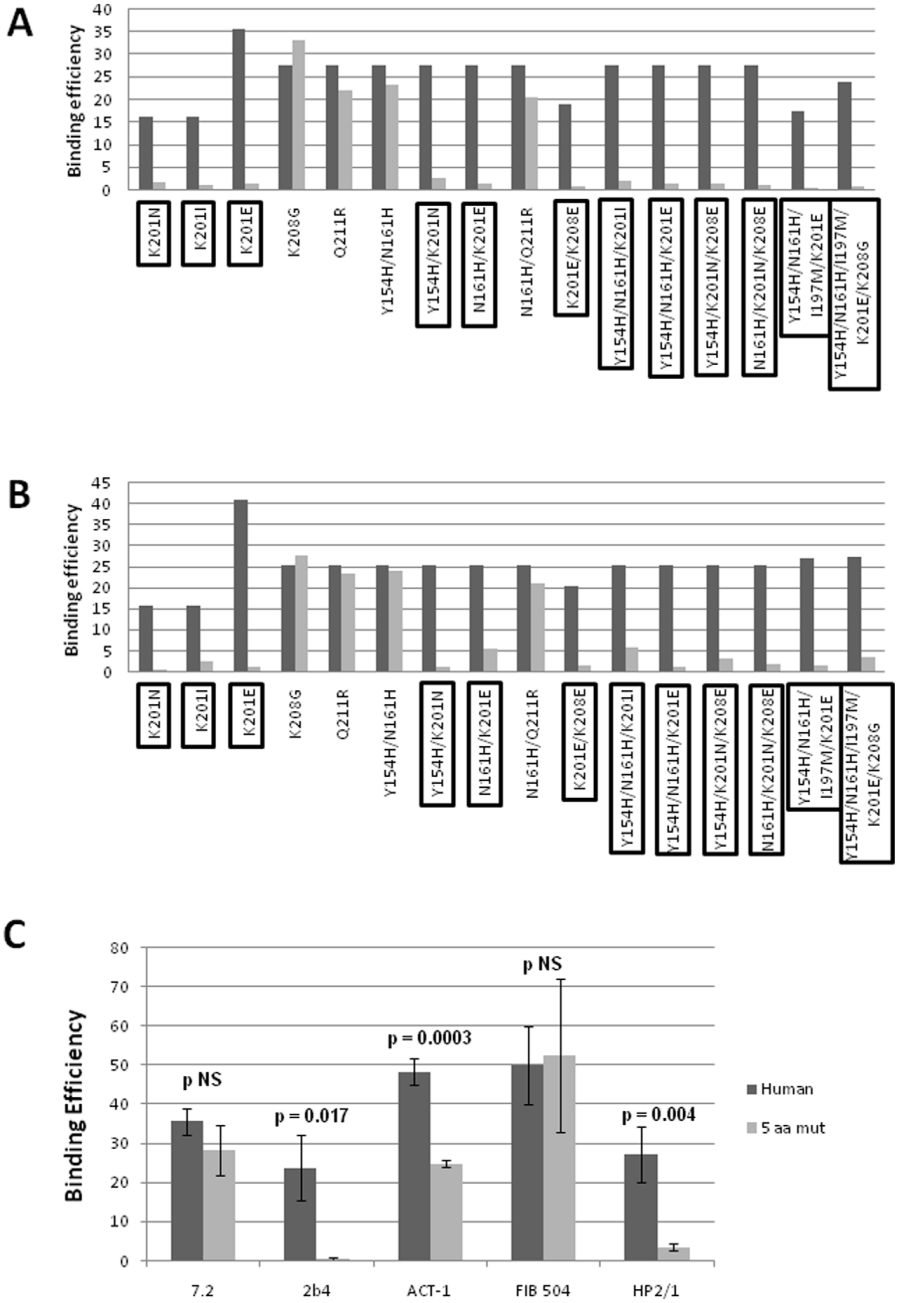
Binding efficiencies (BE) of different α4β7 molecules composed of distinct α4 mutants to monoclonal antibodies against α4, β7 or the α4β7 heterodimer. Binding efficiency is determined by the ratio between the mean fluorescence of antibody binding to each α4 molecule and of the binding in a mock-transfected cell culture (see Materials and Methods for details). Dark gray bars represent binding to the human (wild type) α4 clone, whereas light gray bars are those of binding to the different α4 mutants (as shown in the *x*-axis). α4 mutants which included substitutions at codon 201 are boxed. ***A***, binding of anti-α4 2b4 antibody. ***B***, binding of anti-α4 HP2/1 antibody. ***C***, BE of different anti-α4 and β7 antibodies to the human α4 and the quintuple α4 mutant (5 aa mut). Bars represent the range of standard errors deduced from triplicate experiments. *p*-values of Student's *t* tests are shown above each comparison. *NS*, non-significant (> 0.05).

Based on the polymorphisms found in *ITGA4* genes from NWP, we determined to dissect the relative impact of each α4 amino acid change on the binding efficiencies of α4-directed monoclonal antibodies and of α4 natural ligands. To pursue this, we constructed a panel of single and multiple mutant α4 variants, with different combinations of the polymorphisms displayed by NWP specimens (Table 2), and tested their ability to bind to those molecules. Figure 3 depicts the results of the binding assays to anti-α4 mAbs 2b4 and HP2/1. A plethora of different binding efficiencies (BE) were observed when testing all α4 mutants, but, in general the effects of these mutants were similar for both 2b4 and HP2/1 (compare Figures 3A and B). This is consistent with the idea that both mAbs target closely space epitopes in the same region of α4. It is noteworthy that this is the same region of α4 that mediates binding to the α4β7 natural ligands MAdCAM-1 and VCAM-1.[10] The anti-α4 antibody 7.2 (which targets a distal region of α4) [10] and the anti-β7 antibody, FIB504, did not show differences in binding to α4β7 molecules that incorporated the NWP polymorphisms relative to wild-type α4β7 (data not shown). Most strikingly, all single and multiple mutants containing changes at codon 201 (highlighted in both Figures 3A and B), irrespective of the amino acid residue change, had a strongly reduced binding to both HP2/1 and 2b4. Substitution with either non-polar or polar (negative) amino acid residues disrupted binding of both mAbs. These results suggest that residue 201 plays a critical role in the binding of both of these antibodies to α4. We carried out in triplicate experiments comparing the wild-type human *ITGA4* gene with the quintuple mutant Y154H/N161H/I197M/ K201I/K208G, which contains the most frequent polymorphisms found among Platyrrhini, and is characteristic of the *Callithrix*, *Cebuella* and *Mico* genera. As depicted in Figure 3C, the BE of mAbs 2b4, HP2/1 (both anti-α4), and of ACT-1 (anti-α4β7) to this mutant α4β7 was significantly reduced when compared to wild-type α4β7. Again, no differences in binding efficiencies were observed for the anti-α4 7.2 and the anti-β7 FIB504 antibodies (Figure 3C). From the 7.2 and FIB504 results we can conclude that mutant and wild type heterodimers were expressed at similar levels in our system, and therefore the differences observed are rather explained by distinct differences in affinities of the α4 variants for 2b4 and HP2/1.

**Figure 4:**
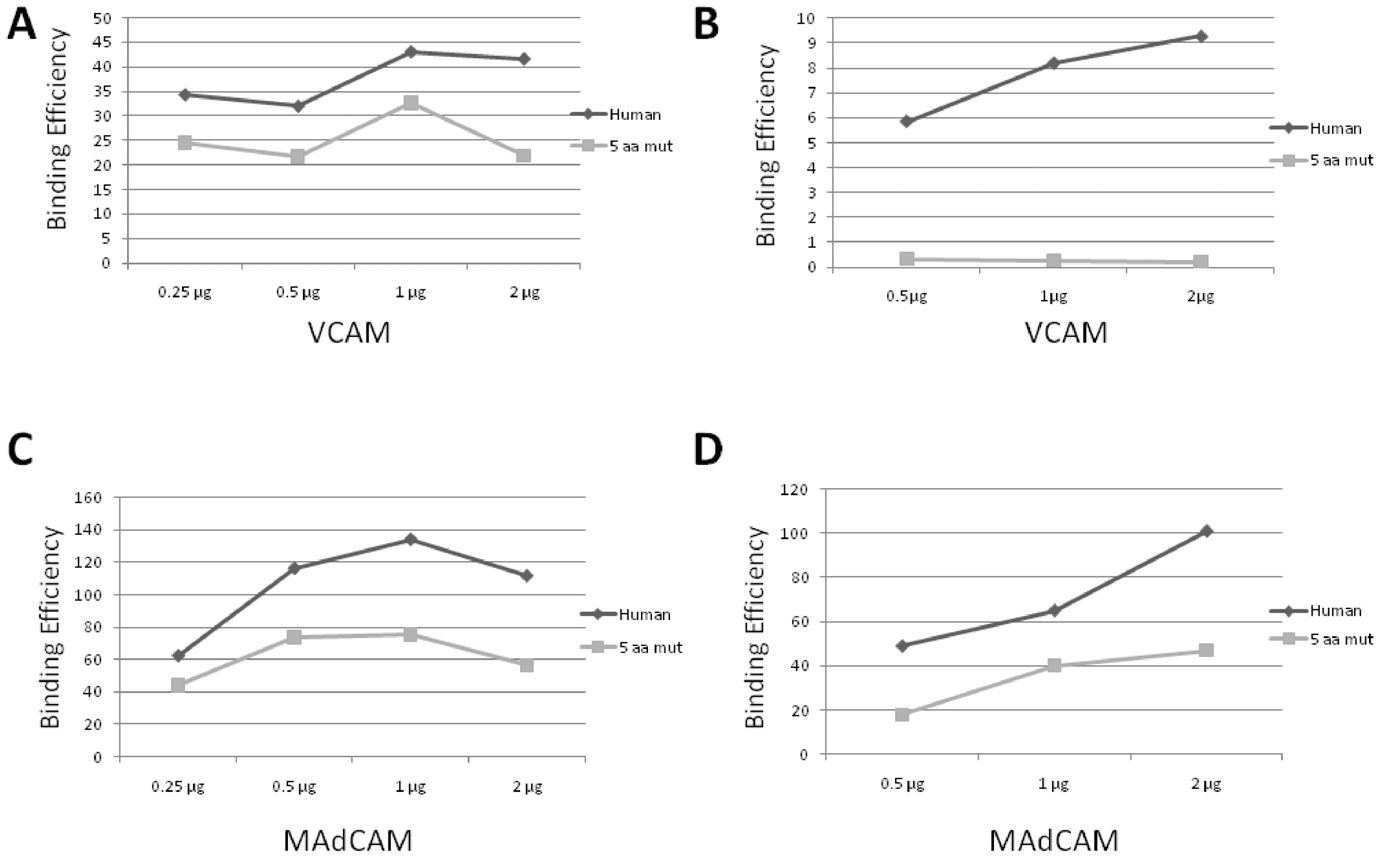
Binding efficiencies (BE) of VCAM (*A* and *B*) and MAdCAM (*C* and *D*) to the human α4 and the quintuple α4 mutant (5 aa mut) in the presence of Mn^2+^ (*A* and *C*) and Mg^2+^ (*B* and *D*). Ligands were tested at different amounts (0.25 to 2 µg).

The quintuple mutant was also compared with the human α4 in regard to the binding to α4β7 natural ligands, VCAM and MAdCAM. 293T cells expressing recombinant α4β7 proteins were incubated with biotinylated VCAM- or MAdCAM-Ig fusion proteins and their binding was monitored by flow cytometry. We have conducted these assays in two different conditions, in the presence of a buffer containing Mn^2+^ or Mg^2+^. While Mn^2+^ increases the steady-state affinity of the natural ligands VCAM and MAdCAM to α4β7 [30], Mg^2+^ may better mimic *in vivo* physiological conditions. As expected, there was a sharp decrease in VCAM interactions with both wild-type and α4 variant when tested in the presence of Mg^2+^ at different concentrations (from 0.25 to 2 µg; compare Figures 4A and B) relative to binding in the presence of Mn^2+^, consistent with the idea that in the presence of Mg^2+^, α4β7-ligand interactions are less stable and exhibit a lower overall affinity [30]. Strikingly, the magnitude of this decrease was much higher for the mutant α4β7 compared to the wild-type human counterpart (Figure 4B). This demonstrates that the polymorphisms present in the mutant α4β7 have a significant impact on VCAM binding under physiological conditions. Interestingly, MAdCAM did not show the same pattern. Although MadCAM-Ig showed reduced binding to α4β7 in the presence of Mg^2+^ compared to Mn^2+^ (compare Figures 4C and D), the relative effect of the mutations was less pronounced. This is consistent with the notion that MAdCAM, because it is able to engage both the intermediate- and high-affinity conformations of α4β7, is less sensitive to changes mediated by the polymorphisms described in this report. Overall, these experiments corroborate those conducted with the monoclonal antibodies, indicating that the polymorphisms we observed in NWP localize to a functionally relevant domain of α4, and therefore have the potential to disrupt the interaction between α4β7 with its natural ligands [10].

### Binding properties of NWP α4 variants to HIV-1 gp120

**Figure 5:**
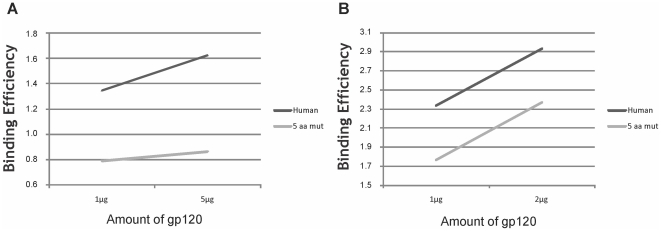
Binding efficiencies (BE) of different recombinant HIV-1 gp120 molecules at different amounts to the human α4 and the quintuple α4 mutant (5 aa mut). gp120 molecules tested were ***A***, AN1 N201Q, subtype B isolate AN1 with a substitution at codon 201 [28]; ***B***, 93MW959, subtype C reference isolate from Malawi (GenBank acc. no. AY713413).

We wanted next to test the ability of neotropical primate α4β7 proteins to bind gp120 molecules from diverse HIV-1 reference and clinical isolates of different genetic subtypes. Recombinant gp120 s were expressed and conjugated to biotin [4], and tested for binding into mutant or human α4β7-expressing 293T cells. Figure 5 depicts BE's of two gp120 s belonging to distinct HIV-1 subtypes (B and C) to the quintuple mutant or to the human α4 allele. The mutant integrin showed slightly reduced binding to both gp120 molecules at two different concentrations in a dose-dependent fashion when compared to the human protein. Although differences in binding of gp120 were observed for the α4 alleles, they were modest compared to those seen for monoclonal antibodies and natural ligands such as VCAM and MAdCAM. This suggests that the binding site of gp120 to α4β7, although similar, is not identical to that of MadCAM and VCAM, and might be determined by additional or more complex protein interactions. The results presented here suggest that HIV-1 viruses of different subtypes have moderately reduced affinity to α4β7 from neotropical primates compared to the human counterpart.

## Discussion

In this study, we have provided evidence that different alleles of *ITGA4* gene, encoding for the α4 subunit of α4β7 integrin in primates, have disparate abilities to bind to α4β7 natural ligands, anti-α4β7 monoclonal antibodies, and to lentiviral gp120 envelope proteins. Such differences in binding appear to be governed by amino acid residue changes in the α4 protein sequences, which have arisen and have been fixed during primate evolution.

A multitude of *ITGA4* genotypes were found by sequencing exons 5 and 6 of the gene in neotropical primates. This region of the gene was chosen in view of its well described functional importance in mediating the binding of α4β7 to its natural ligands VCAM and MAdCAM [10], as well to certain monoclonal antibodies [10] and to the gp120 envelope protein of HIV-1 [4]. Most of the changes observed appeared to be lineage-specific, being characteristic of distinct Platyrrhini species, genera or families. In general, these polymorphisms appeared to follow the radiation of this primate group, and their emergence could be traced in the phylogeny of the infraorder (Figure 1). Whereas most of the amino acid changes observed were conservative, several resulted in a change of local protein net charge and might have a significant influence on structure. This was the case of the K201N/I/E and K208E/G substitutions, found in many distinct genera of NWP.

The analysis of each polymorphism on an individual basis, as well as in combinations of two, three, four and five changes, has lead us to dissect their particular role on the binding of mAbs, natural ligands (VCAM and MAdCAM) and HIV-1 gp120 proteins to α4β7. As a consequence, we were able to pinpoint the specific role of residue 201 of α4 on the binding of all these molecules. All mutants harboring non-conservative changes at that α4 codon, either alone or in combination with other polymorphisms, showed reduced affinity to some or all of the molecules tested. These included mutants with changes of the original lysine residue found in humans to an isoleucine, an asparagine or a glutamic acid, found in distinct NWP genera.

We have observed varying effects of the α4 polymorphisms when assessing binding of different ligands, such as monoclonal antibodies, natural ligands (VCAM and MAdCAM) and HIV-1 gp120 molecules. The most dramatic effects on binding were seen for the mAbs that target the functional motifs of α4. Of note, a significant obliteration was observed for HP2/1 binding, an antibody that was used as a basis for the development of natalizumab, a clinically approved and widely used drug for the treatment of neurodegenerative disorders such as multiple sclerosis [31], [32], and under clinical trials for Crohn's disease and other autoimmune conditions [33], [34]. Although we have not yet identified a similar polymorphism in human α4, we can envisage a scenario in which particular *ITGA4* polymorphisms in humans might negatively impact the efficacy of natalizumab for treating neurological disorders as well as other autoimmune diseases.

We have also found pronounced effects of VCAM binding to the mutant α4β7, particularly when our assay conditions mimicked physiological conditions such that a significant fraction of α4β7 was presented on the cell surface in an intermediate affinity conformation. Under these conditions, VCAM binding to α4 variants was completely disrupted. This observation suggests that these variants could hold the potential to compromise the biological function of α4β7 and α4β1 integrins. It will be of interest to assess certain immunological processes, including leucocyte trafficking, the formation of immunological synapses and cellular immune responses [35] in neotropical primates bearing these polymorphisms. Additionally, the study of polymorphisms in VCAM and MAdCAM present in those animals is also worthwhile, since compensatory substitutions in those proteins may have arisen during NWP evolution to counteract the observed variations in α4.

A mutant α4β7 carrying K201N/I/E, again alone or in combination with other α4β7 polymorphisms, also showed reduced affinity to HIV-1 gp120 proteins of different subtypes. Although the changes in binding to the mutant α4β7 were the most modest observed, in our preliminary analyses we only tested gp120 binding in the context of high α4β7 activation (in the presence of Mn^2+^). It is possible that under more physiological conditions (Mg^2+^), such reduced binding phenotype is even more pronounced, as it has been observed for VCAM binding. Moreover, we have only tested a limited number of gp120 molecules, and the reactivity of gp120 s for α4β7 varies over a wide range [28], so that the influence of the α4 polymorphisms may be different with other gp120 s. Further studies are needed to address this issue. Finally, it is worth mentioning that our test only assesses an *in vitro* binding ability, and it is conceivable that under a natural transmission scenario, followed by multiple rounds of virus replication, the differences observed may indeed relate to more pronounced effects on HIV acquisition or on disease progression. We are just starting to unveil the molecular interactions between gp120 and α4β7, as it may be as complex as the interaction with natural ligands MAdCAM and VCAM [36]. Additional studies characterizing such interaction are ongoing.

We hypothesize that α4β7 may be considered an additional factor restricting lentiviral infections in neotropical primates, similarly to what has been described for other lentiviral interacting cellular proteins such as the chemokine receptors CCR5 and CXCR4 [16], [17], TRIM5α [19], [20], [22], the APOBEC proteins [23], [24] and tetherin [25]. In fact, the reduction of HIV-1 gp120 binding to α4β7 harboring K201E/I/N, in which the substitution of a positively-charged amino acid for a neutral or even negatively charged residue occurs, can be mechanistically compared to the binding of gp120 to the coreceptors CCR5 or CXCR4 through its V3 loop. In that instance, the net charge of amino acids 11 and 25 of the V3 loop sequence determine a higher affinity of the protein to either CXCR4 (when the net charge is positive) or to CCR5 (when it is negative), in a phenomenon often referred to as the “11/25 rule” [37]. We hypothesize that in the interaction studied herein, that of HIV-1 gp120 binding to α4β7, the net charge of α4β7 residue 201 may also be crucial for the binding of the viral envelope protein to this newly characterized receptor. Further studies are necessary to evaluate the relative binding efficiencies of gp120 to distinct NWP α4 variants carrying distinct amino acid residues at position 201 (see Figure 2 for details).

A multitude of NWP *ITGA4* phenotypes may be extrapolated from their amino acid diversity. Similar disparities in lentivirus restriction phenotypes have been described in NWP for other restriction factors such as APOBEC3, TRIM5α and tetherin proteins [19], [21], [24], [25]. As a result, NWP cells may modulate viral restriction activities through a complex balance of different protein factors. Of note, owl monkeys (*Aotus*) have been shown to carry a TRIM-Cyp fusion protein that potently restricts HIV-1 [21]. On the other hand, these monkeys possess a polymorphism in tetherin which renders it inactive against HIV-1 [25]. In our study, owl monkeys were shown to harbor a minority variant α4 protein similar to the human counterpart (harboring a lysine at residue 201). We can depict a scenario in which different primates have developed alternative strategies for counteracting retroviral infections. The antiviral activity of other NWP carrying a lysine at that position (such as *Leontopithecus*, *Saguinus* and members of the Piithecidae family) is warranted further investigation.

A relatively high genetic diversity was found in the *ITGA4* gene of neotropical primates, with some alleles displaying reduced binding to mAbs, natural ligands and HIV-1 gp120. The existence of *ITGA4* polymorphisms in other primate groups, including those of African and Asian origin, is anticipated. Therefore, it is conceivable that *ITGA4* polymorphisms in primate species which are susceptible to, or even natural reservoirs of lentiviral infections (such as those carried out by SIV or HIV) display a multitude of lentivirus restriction phenotypes. Clinical and laboratory outcomes such as lentivirus acquisition, resistance to disease, disease progression, control of viremia and CD4^+^ T-cell depletion are among the phenomena that may be influenced by *ITGA4* polymorphisms in those primate species, including humans, and their study is warranted further attention. Moreover, additional studies are required to fully appreciate the consequences of *ITGA4* polymorphisms in the development and treatment of infectious and autoimmune diseases in humans and in their non-human primate models.

## Supporting Information

Table S1 **List of primers used for the construction of mutant α4 clones.**
(DOC)
