# Supplementary material for: Polymorphisms in the α4 Integrin of Neotropical Primates: Insights for Binding of Natural Ligands and HIV-1 gp120 to the Human α4β7
Source: PLoS One. 2011 Sep 2;6(9):e24461. doi: 10.1371/journal.pone.0024461 (PMC3166318; doi:10.1371/journal.pone.0024461)
Supplement: Table S1 — List of primers used for the construction of mutant α4 clones. (DOC) [file pone.0024461.s001.doc]

**Supplementary** **Table S1**. List of primers used for the construction of mutant α4 clones

| *ITGA4* | Polymorphism | Primer position^a^ | Primers^b^ | Tm^c^ |
| --- | --- | --- | --- | --- |
| Exon 5 | Y154H | 548 – 568 | F – 5’ GTTATCAAGAT**C**ATGTGAAA 3’  R – 5’ TTTCACAT**G**ATCTTGATAAC 3’ | 45.3ºC |
|  | N161H | 573 – 593 | F – 5’ TGGAGAA**C**ATTTTGCATCATG 3’  R – 5’ CATGATGCAAAAT**G**TTCTCCA 3’ | 51.5ºC |
| Exon 6 | I197M | 679 – 699 | F – 5’ GTCTACAATAT**G**ACTACAAAT 3’  R – 5’ ATTTGTAGT**C**ATATTGTAGAC 3’ | 44.9ºC |
|  | K201I | 691 – 710 | F – 5’ ACTACAAATA**T**ATACAAGGC 3’  R – 5’ GCCTTGTAT**A**TATTTGTAGT 3’ | 44.3ºC |
|  | K201E | 691 – 710 | F – 5’ ACTACAAAT**G**AATACAAGGC 3’  R – 5’ GCCTTGTATT**C**ATTTGTAGT 3’ | 47.9ºC |
|  | K201N | 691 – 710 | F – 5’ ACTACAAATAA**C**TACAAGGC 3’  R – 5’ GCCTTGTA**G**TTATTTGTAGT 3’ | 47.3ºC |
|  | K208E | 716 – 733 | F – 5’ TAGAC**G**AACAAAATCAAG 3’  R – 5’ CTTGATTTTGTT**C**GTCTA 3’ | 44.2ºC |
|  | K208G | 708 – 743 | F – 5’ GGCTTTTTTAGAC**GG**ACAAAATCAAGTAAAATTTGG 3’  R – 5’ CCAAATTTTACTTGATTTTGT**CC**GTCTAAAAAAGCC 3’ | 60.0ºC |
|  | Q211R | 720 – 738 | F – 5’ CAAACAAAATC**G**AGTAAAA 3’  R – 5’ TTTTACT**C**GATTTTGTTTG 3’ | 43.6ºC |

^a^ Position of primers according to the *ITGA4* cDNA sequence (Gene ID 3676)

^b^ Nucleotides in bold and underline are those introduced by site-directed mutagenesis

^c^ Tm, annealing temperature used in the mutagenesis PCR
